# Editorial: Women in infectious agents and disease: 2024

**DOI:** 10.3389/fmicb.2025.1694902

**Published:** 2025-09-23

**Authors:** Alina Maria Holban, Nayeli Alva-Murillo, Svetlana Khaiboullina, Ze Chen

**Affiliations:** ^1^Department of Botany and Microbiology, University of Bucharest, Bucharest, Romania; ^2^Department of Biology, University of Guanajuato, Guanajuato, Mexico; ^3^University of Nevada, Reno, Reno, NV, United States; ^4^Department of Biochemistry and Molecular Biology, Hebei Normal University, Shijiazhuang, China

**Keywords:** infectious disease, women, microbes, pathogenesis, diagnosis

Only a third of the researchers in the science, technology, engineering, and mathematics (STEM) fields are women. Women remain underrepresented in many fields of science, including Microbiology. The higher rate of male compared to female corresponding authors in manuscripts published in the American Society for Microbiology (ASM) journal was shown by Hagan et al. (2020). Also, only 18% of Editors-in-Chief of all ASM journals were women. Additionally, authors indicate that the outcome of submission is less favorable for women than for men. In another study, Nelson and Rogers (2003) indicate that the representation of women drops from 50% receiving the doctorate degree to 30% holding tenure-track positions. This makes women less likely to obtain the grant funding for the research, which could impact their research career success. Our Research Topic aims to address this discrepancy by providing women a platform for publishing high-quality research, which will boost their careers and improve their visibility in the research field.

The present Research Topic, focusing on microbial pathogenesis, includes 12 publications (9 original research articles and 3 reviews). By providing open-access, cutting-edge original research and comprehensive reviews, this Research Topic serves to further support and promote women in microbiology research.

In the original research, Bok et al. addressed the role of the commensal *Escherichia coli* population in the pathogenesis of extraintestinal infections. *E. coli* is the most common pathogen, which can cause a diverse range of diseases (Pitout et al., 2015). This bacterium can cause bacteremia in older adults and urinary tract infections in young women (Nguyen and Götz, 2016; Yahav et al., 2016). Also, *E. coli* is a frequent cause of meningitis in neonates (Kim, 2016). Extraintestinal *E. coli* infection morbidity and mortality rates are increasing worldwide (Rosenberg et al., 2021; Russo and Johnson, 2003). Also, the emergence of multidrug-resistant *E. coli* represents a significant challenge for healthcare providers (Ohmagari et al., 2023). However, there are still gaps in our understanding of the genetic and phenotypic variations of *E. coli* associated with extraintestinal infections. In this study, genotypic and phenotypic characteristics of two commensal *E. coli* isolates from adults and children were examined. A higher frequency of pathogenicity islands and virulence genes was found in isolates from adults compared to those from children. The dominant phylogroup was B2, with sequence types ST73, ST59, ST131, ST95, ST141, and ST69 being more prevalent in adult isolates. Conversely, a different subset, ST10, ST155, ST59, and ST1823, was found in isolates from children. Crucially, the hemolytic activity and siderophore receptor expression were more common in these adult-derived isolates. Traits not associated with phylogroup B2, such as adhesion abilities mediated by type 1 and P fimbriae and biofilm formation propensities, were similar in isolates from both adults and children. The importance of monitoring the genetic and phenotypic profiles of commensal *E. coli* was indicated.

Another original research by Chen et al. evaluated Human papillomavirus (HPV) competition in unvaccinated women. Human papillomavirus infection is linked to 90% of cervical cancer (WHO, 2024b). The incidence and mortality rates remain on the rise worldwide. According to the WHO, there were 660,000 new cases diagnosed in 2022, with around 350,000 fatalities (WHO, 2024a). There are several vaccines available on the market to prevent HPV infection (Cheng et al., 2020). These vaccines target HPV-16, 18, 6, and 11, which are responsible for most cervical cancer cases (Petrosky et al., 2015). It should be noted that the vaccine can prevent infection with the targeted HPV; however, commonly used vaccines do not target all high-risk HPV types. Therefore, there is a concern of the “type replacement after HPV vaccination (Man et al., 2019). The “type replacement” refers to an increased infection rate of certain HPV types due to the elimination of vaccine-targeted HPV types (Tota et al., 2017). The Authors addressed the “type replacement” concern in this study. Of 159,049 women, 19.8% tested positive for HPV, with 5.1% exhibiting multiple type infections. Significant negative associations were observed between HPV-6 and HPV-72, HPV-18 and HPV-72, HPV-31 and HPV-83, HPV-33 and HPV-26, HPV-45 and HPV-55, HPV-56, and HPV-26, as well as HPV-59 and HPV-69, suggesting potential type competition. However, no type competition pair was found in the cohort study. Conversely, women with vaccine-targeted types had a higher risk of acquiring other types (HR > 1.0).

The study by Diaz-Navarro et al. assessed the association between recurrent vulvovaginal candidiasis (RVVC), biofilm-forming capacity, and clinical symptoms. RVVC can be attributed to an imbalance between *Candida* spp. and vaginal biota, which could be caused by long-term antibiotic treatment, age of the patients and use of contraceptives (Díaz-Navarro et al., 2023). Also, *Candida* spp. virulence factors could contribute to the adhesion of the microbe to the vaginal epithelium (Kalia et al., 2020; Li et al., 2022). This multifactorial nature of pathogenesis makes the treatment of RCCV challenging. Improving our understanding of the role of these factors in the pathogenesis of RCCV will facilitate the selection of the best treatment for the disease. A total of 271 patients were positive for *Candida* spp. in vaginal swabs. During 1 year of observation, 55 (20.3%) patients experienced at least one recurrence, with 19 (7.0%) meeting the criteria for RVVC (≥3 episodes/year), accounting for 65 episodes in total. Most isolates were *Candida albicans* (90.0%). The authors concluded that RVVC had high biomass production. Additionally, RVVC clinical isolates tended lower metabolic activity, which may contribute to treatment failure.

An original work by Gohain et al. aimed to identify targets against *Streptococcus pneumoniae* (*S. pneumoniae*) using an *in silico* subtractive genomics approach. *S. pneumoniae* is a significant community health concern. This bacterium can cause meningitis, pneumonia and bacteriemia, characterized by high morbidity and mortality rates (Shami et al., 2023). Antibiotic resistance of *S. pneumoniae* is on the rise and reported globally (Cillóniz et al., 2018; Cools et al., 2021). This has become a significant health care issue and poses an urgent need for new treatments for drug-resistant *S. pneumoniae*. 2,000 out of the 2,027 proteins from *S. pneumoniae* were non-homologous to human proteins. After screening against the Database of Essential Genes (DEG) and human gut microflora, 21 essential genes were prioritized, with Key hub genes such as *gpi, fba, rpoD*, and *trpS* associated with 20 pathways. Screening of 2,509 FDA-approved compounds identified Bromfenac as a leading candidate, exhibiting a binding energy of −26.335 ± 29.105 kJ/mol. Bromfenac, particularly when conjugated with AuAgCu_2_O nanoparticles, has demonstrated antibacterial and anti-inflammatory properties against *Staphylococcus aureus*. This suggests that Bromfenac could be repurposed as a potential therapeutic agent against *S. pneumoniae*, pending further experimental validation.

The original research conducted by Aswal et al. focused on identifying pathogenicity proteins in *Escherichia coli*. Multidrug-resistant (MDR) *E. coli* can cause high morbidity and mortality (Daneman et al., 2023). Microbial drug resistance is a complex phenomenon influenced by an interplay of several genomic, transcriptomic and proteomic factors. Through integrated RNA-Seq and SWATH-LC MS/MS analyses, 763 genes and proteins were found to have differential expression. Of these, 52 genes showed differential expression in MDR *E. coli*. The majority of these proteins were associated with secondary metabolites, aminoacyl-tRNAs and ribosomes. Among these, multiple hub proteins were involved in aminoacylation of tRNA, lysyl-tRNA and translation. Three hub proteins were involved in several biological pathways directly and/or indirectly related to antibiotic stress. The authors suggest that these proteins could serve as potential future therapeutic targets for the treatment of MDR *E. coli*.

The study by Kukovica et al. aimed to determine the efficacy of self-collection of rectovaginal swabs as a valid alternative to collection by healthcare workers (HCWs) during pregnancy. Group B streptococcus (GBS) was found to be associated with up to 50% of invasive infections in newborns (Madrid et al., 2017). Prevention of GBS is the most effective approach to protect newborns, which could be done by the collection of intravaginal and colorectal swabs during the third trimester of pregnancy (Verani et al., 2010). However, limitations in healthcare resources and the COVID-19 pandemic made it challenging to collect swabs by medical personnel. Self-collection has emerged as a complementary approach to better accessibility, convenience, and privacy (Smith et al., 2024; WHO, 2024c). This approach could also improve compliance with screening programs. The authors analyzed the efficacy of GBS isolation in samples collected by patients and by HCWs. The authors reported that GBS was detected in 18% collected by HCWs, whereas the self-collection method yielded a GBS positivity rate of 19%. It was concluded that self-collection had a trend for a higher diagnostic yield. It was demonstrated that the most sensitive method was PCR after enrichment from self-collected samples. The authors concluded that self-collection of rectovaginal swabs during pregnancy could be an alternative to collection by HCWs. Also, PCR from enrichment showed better detection of GBS compared to enrichment culture.

Original research by Ali et al. focused on demonstrating that isothermal recombinase DNA amplification coupled with lateral flow (LF) chromatography on a PCRD cassette is a simple and rapid molecular diagnostic test to detect and identify *Leishmania major*. Cutaneous leishmaniasis (CL) can cause disfiguring lesions, scars and psychological distress and social stigma (Chahed et al., 2016). The diagnosis is based on microscopic direct examination of Giemsa-stained smears (Khademvatan et al., 2011). This approach is laborious, time-consuming and requires highly trained and experienced personnel. Microscopic examination has low sensitivity and cannot be used to identify *Leishmania* spp. Currently, only DNA assays accurately identify *Leishmania* spp. (Salotra et al., 2001). These methods require equipment and trained personnel. The authors developed an *L. major* species-specific RPA-LF assay and computational analysis. This novel assay was tested using 86 human cutaneous samples, demonstrating 100% specificity.

Nicholson and Shore studied whether *Bordetella bronchiseptica* (*B. bronchiseptica*) could contribute to the transfer of antimicrobial resistance genes in the swine production environment. *B. bronchiseptica* is a highly contagious respiratory pathogen (Vötsch et al., 2021). This bacterium has a broad host range, including wild and domestic mammals (Miguelena Chamorro et al., 2023). *B. bronchiseptica* increases colonization of the respiratory tract with *Glaesserella parasuis, Pasteurella multocida*, and *Streptococcus* in swine (Loving et al., 2010; Vötsch et al., 2021). Some of these co-colonizing microbes could harbor antimicrobial resistance genes (Hau et al., 2018; Nicholson and Bayles, 2022), which could be transferred to *B. bronchiseptica*. Therefore, it is important to analyze the genetic diversity and antimicrobial resistance genes in *B. bronchiseptica* isolates from swine. The authors identified a high degree of genomic conservation in swine *B. bronchiseptica* isolates. The majority of *B. bronchiseptica* isolates had resistance to four antibiotic classes. However, only three antimicrobial resistance genes were identified. Data suggest that *B. bronchiseptica* isolates are not serving as a source of antimicrobial resistance gene transference.

Gao et al. investigated the incidence, clinical characteristics, risk factors, microbiological features, and antibiotic resistance patterns of maternal peripartum bloodstream infection (BSI). Over half of intrahospital death is accounted for by infection-related maternal deaths, according to the Global Maternal Sepsis Study group (Bonet et al., 2020). Maternal BSI remains a significant health care problem characterized by high morbidity and mortality (Surgers et al., 2013; Zou et al., 2021). However, our understanding of the microbiological characteristics, clinical features, and prognoses is limited. This study focused on understanding the roles of premature rupture of membranes (PROM) and fever in BSI. Authors included BSI (*n* = 85) and non-BSI (*n* = 361) groups. It was demonstrated that spontaneous rupture of membranes (PROM, vaginal examinations >5 times, and cesarean sections during labor were more common in the BSI group. *Escherichia coli* (58.1%) was the major causative pathogen, followed by *Enterococcus faecalis* (7.1%). The BSI group exhibited higher rates of maternal sepsis and Apgar scores ≤ 7 at 1 min. Furthermore, PROM, fever ≥38.9 °C (102°F), and fever within 24 h after delivery were risk factors for postpartum BSI in the adjusted analysis. The authors conclude that the maternal BSI is associated with PROM. Early identification of pathogens and antimicrobial resistance can prevent adverse outcomes.

In the review, Huang et al. summarized diagnostic methods for Bovine rotavirus (BRV), discussed their advantages, and presented future perspectives on BRV diagnosis. This review aims to provide references for the effective diagnosis and control of BRV-related diseases. BRV can cause diarrhea in calves, profoundly impacting the cattle industry, resulting in substantial economic losses (Geletu et al., 2021). The diagnostic approaches for BRV primarily include etiological methods, such as electron microscopy, virus isolation, and culture (Cho and Yoon, 2014). Serological methods such as enzyme-linked immunosorbent assay (ELISA), latex agglutination test (LAT), and immunofluorescence techniques are widely utilized for BRV diagnosis. Additionally, molecular methods, including reverse transcription-polymerase chain reaction (RT-PCR), real-time quantitative PCR (qPCR), and loop-mediated isothermal amplification (LAMP), as well as next-generation sequencing (NGS) technology, are also used to diagnose BRV (Irehan et al., 2025).

The systematic review by Ji et al. compared the accuracy and acceptance of self-sampling to clinician sampling for Human papillomavirus (HPV) testing in Asia. The authors included 67 studies, revealing that the sensitivity and specificity of HPV self-sampling for detecting cervical intraepithelial neoplasia were higher than 80% and 70%, respectively, consistent with clinician sampling results. The consistency between self-sampling and clinician-sampling was high in most studies, with kappa values exceeding 0.7. Women had high acceptance of self-sampling but expressed some concerns. The authors conclude that self-sampling for HPV testing can significantly improve cervical cancer screening coverage. This is especially important in areas with limited medical resources or reluctance to accept physician sampling. However, the diagnostic criteria and HPV detection methods need to be adjusted due to the low sensitivity of HPV self-sampling in studies from China and India.

The review by Chen et al. addressed the impact of climate change on the spread of severe fever with thrombocytopenia syndrome (SFTS) and its biological vectors. SFTS caused by *Dabie bandavirus*, transmitted by ticks (Kim and Park, 2023). This disease emerged in 2009 in Hubei and Henan provinces of Central China and spread over East and Southeast Asia (Cui et al., 2024). *H. longicornis* tick species are the principal vector for the Dabie bandavirus (Luo et al., 2015). The prevention and control strategies for SFTS include tick' control and public education (Centers for Disease Control Prevention (CDC), 2024). Public health policies also aimed to reduce contact between ticks and animals and alleviate the disease's impact on human health. SFTS is an emerging tick-borne zoonotic disease (USDA, 2024).

In conclusion, this Research Topic highlights a collection of research articles authored by women, focusing on some of the most pressing challenges in infectious disease. These studies explore novel pathogenicity markers of infectious agents, uncover host risk factors, and investigate mechanisms of antibiotic resistance. Collectively, this Research Topic underscores the vital contributions of women to advancing research in the field.
